# Pharmacogenomic analysis indicates potential of 1,5-isoquinolinediol as a universal anti-aging agent for different tissues

**DOI:** 10.18632/oncotarget.3949

**Published:** 2015-04-29

**Authors:** Mi Sung Park, Joon-Seok Choi, Wan Lee, Yoon Jung Yang, Juhee Kim, Gun-Joo Lee, Sang Soo Kim, Seong Hoon Park, Sung Chul Kim, Jin Woo Choi

**Affiliations:** ^1^ Institute for Metabolic Disease, School of Medicine, Wonkwang University, Iksan, Jeonbuk, South Korea; ^2^ College of Pharmacy, Catholic University of Daegu, Gyeongbuk, South Korea; ^3^ Department of Oral and Maxillofacial Radiology, College of Dentistry, Wonkwang University, Jeonbuk, South Korea; ^4^ Wonkwang Institute of Integrative Biomedical Science and Dental Research Institute, School of Dentistry, Wonkwang University, Iksan, Chonbuk, South Korea; ^5^ Jaesaeng Hospital, Biomedical Research Institute, Seongnam, Gyenggi-do, South Korea; ^6^ Division of Cardiothoracic Radiology, Department of Radiology, School Of Medicine, Wonkwang University, Iksan, Jeonbuk, South Korea; ^7^ Department of Acupuncture and Moxibustion, Wonkwang University Oriental Medical Hospital, Gwangju, South Korea; ^8^ Advanced Institute of Convergence Technology, Seoul National University, Suwon, Gyenggi-do, South Korea

**Keywords:** aging, cellular replicative senescence, gene expression profile, chemical genomics

## Abstract

The natural aging of multicellular organisms is marked by a progressive decline in the function of cells and tissues. The accumulation of senescent cells in tissues seems to eventually cause aging of the host. Nevertheless, gene expression that influences aging is unlikely to be conserved between tissues, and age-related loss of function seems to depend on a variety of mechanisms. This is a concern when developing anti-aging drugs in geriatric clinical pharmacology. We have sought a universal agent to redundantly cover gene expression despite the variation in differentially expressed genes between tissues. Using a minimally modified connectivity map, the poly (ADP-ribose) polymerase (PARP) inhibitor 1,5-isoquinolinediol was selected as a potent candidate, simultaneously applicable to various tissues. This choice was validated *in vitro*. Treatment of murine embryonic fibroblasts with 1,5-isoquinolinediol appeared to efficiently suppress the rate of replicative senescence at a concentration of 0.1 μM without resulting in cell death. The appearance of abnormal nuclei and accumulation of β-galactosidase in the cytoplasm were inhibited by daily treatment with the agent. When the aging process was accelerated by hydroxyurea-induced oxidative stress, the effect was even more noticeable. Thus, 1,5-isoquinolinediol may potentially be developed as an agent to prolong life.

## INTRODUCTION

As aging is a main risk factor for many diseases such as vascular and neurodegenerative disorders, understanding the mechanism of aging is critical for maintaining a healthy life and extending the life span [1, 2]. According to the systemic theory, aging is associated with a decline in the function of essential organ systems [3]. There are also several common physiological and pathological characteristics of aging [4], and aging simultaneously affects most tissues. For instance, muscle strength and motor performance are generally reduced with aging, resulting in loss of function [5]. Neurologically, white matter and the length of nerve fibers in the motor cortex are decreased [6], and the ability to transmit information declines accordingly. The aging process is also associated with basic cellular mechanisms such as DNA repair and mitochondrial function [7]. At the molecular level, many genes, including tumor suppressor or pro-apoptotic genes, have been reported as relevant to aging or cellular senescence [7]. However, despite many common genetic alterations in the aging process, the main mechanisms causing aging seem to differ in each tissue [7, 8]. Modern cell biology suggests that most diseases are initiated by the appearance of diseased cells. In this context, cellular replicative senescence is considered to lead to aging of the organism [9, 10]. Baker et al. partially proved this concept with experiments showing that the removal of senescent cells prolonged the life span of mice [9]. However, the relationship between senescent cells and aging in each tissue is not fully understood.

Oxidative and nitrosative stress from reactive oxygen species plays an important role in the aging of endothelial cells [11]. These species affect vascular function as well as endothelial gene expression and the integrity of the monolayer toward senescence [9]. Mitochondria seem to have a more important role for the aging in the muscle than in other tissues [5, 12]. Apoptosis is initiated when mitochondrial function declines and causes energy depletion within the cell. In turn, mitochondrial dysfunction is caused by an accumulation of oxidative damage to macromolecules such as mitochondrial DNA, RNA, and proteins, which are essential for the optimal functioning of mitochondria [13]. Evidence suggests that the molecular mechanism of aging can be different in each tissue. These differences may be one of the reasons for delays in the development of an anti-aging agent.

To date, a few drugs, including rapamycin and resveratrol, have presented positive longevity effects. Resveratrol has been thought of as an activator of sirtuins, a family of deacetylases (including SIRT1) that could promote anti-aging effects without restricting the calorie intake [14-16]. Although recent studies have failed to prove that resveratrol is beneficial for increasing the life span in yeast or mice, it could still be used to access some of the same molecular pathways as those involved in calorie restriction [17]. While resveratrol has not been proven to increase life span, rapamycin has shown much more promise [18]. Rapamycin has been shown to increase longevity by inhibiting the target of rapamycin kinase (TOR) through an effect similar to calorie restriction [19]. Testing in model systems that included yeast, nematodes, flies, and mammals has shown that a reduction in TOR signaling will result in an increased life span [14, 19].

Despite the few drugs listed above, there is still a need to develop more drugs to increase longevity. A drug that can broadly target different genetic alterations in various tissues during the aging process is particularly desirable. In order to find an agent to cover several tissues, we utilized a connectivity map, a recently developed tool to investigate connections among small molecules that share a mechanism of action, chemical and physiological processes, and diseases and drugs [20]. This algorithm predicted common agents that would cover the aging-related gene signatures obtained from three different tissues. A poly (ADP-ribose) polymerase (PARP1) inhibitor was finally isolated as a drug that can be applied effectively on genetically diverse tissues. Through *in vitro* validation, we propose 1,5-isoquinolinediol as a universal anti-aging agent to suppress senescence processes that are occurring simultaneously in many tissues.

## RESULTS

### Systematic analysis of aging-associated gene expression profiles from the muscle, artery, and frontal gyrus of the brain

**Figure 1:**
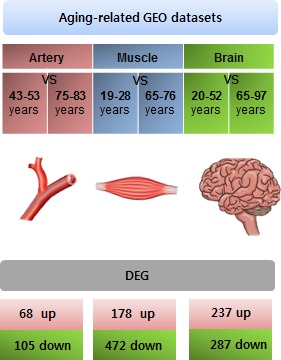
Collection of aging-related gene expression signatures from the artery, muscle, and brain Schematic to explain the overall experimental process. To reveal more significant aging-related gene expression, we rearranged the subjects and excluded individuals who were middle-aged. The GEO dataset GSE16487 was used to analyze differentially expressed genes (DEGs) in the aging artery (younger: *n* = 6, older: *n* = 7). GSE38718 was used to analyze genes related to the aging muscle (younger: *n* = 7, older: *n* = 4) GSE11882 was used to analyze DEGs in the aging brain (younger: *n* = 12, older: *n* = 11). DEGs were analyzed by comparing expression between young and older subjects. The DEGs were sorted using GE2R.

Based on previously reported data on aging of the artery (artery media), muscle (biceps brachii muscles), and brain (superior frontal gyrus), we rearranged the gene expression profiles from the GEO datasets for the artery (GSE16487), muscle (GSE38718), and brain (GSE11882) to elicit obvious polarity between young and old tissues. When the gene expression profiles were statistically compared with thresholds of *p* < 0.05 and a 1.5-fold difference, 68 genes were found to be up-regulated and 105 genes were down-regulated in the arteries. In the muscle, 178 genes were up-regulated and 472 genes were down-regulated. In the brain, 237 genes were up-regulated and 287 genes were down-regulated (Figure 1, Figure S1-3 and Data S1).

**Figure 2:**
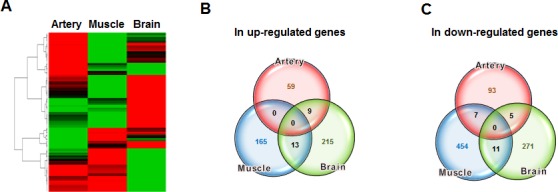
Comparison of aging-related gene expression between different tissues **A.** The DEGs from the artery, muscle, and brain tissues were expressed in parallel in the form of a heat map. **B.** The number of up-regulated genes in each group and the number common to the groups were depicted in a Venn diagram. **C.** The number of down-regulated genes in each group was also expressed in a Venn diagram.

The differentially expressed genes (DEGs) were listed in parallel among the three different tissues. The DEGs were divided into 2 groups, designated up and down, according to the direction of the change in expression (Figure 1 lower panel). The DEGs were then expressed as a heat map (Figure 2A) and as Venn diagrams (Figure 2B and 2C). Although the tissues were analyzed based on the aging process, no genes were commonly either up-regulated or down-regulated in all three tissues (Figure 2B and 2C, respectively).

### Connectivity map-based prediction of agents to cover gene expression profiles among different tissues

**Figure 3:**
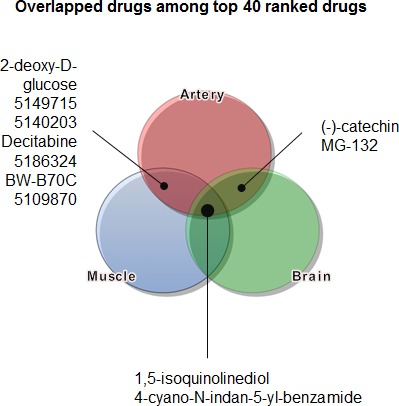
Identification of candidate agents to overcome aging using a connectivity map The DEGs deduced from Figure 2 were used as an input list to apply to a connectivity map. Candidate agents that were common to several tissues were expressed in a Venn diagram.

Each DEG list from the three tissues was submitted to connectivity mapping. From the names of agents obtained for each tissue, based on enrichment score, the top 40-ranked genes were listed (Table S1–3), and chemicals that had an effect on these genes in more than one tissue were depicted in a Venn diagram (Figure 3). Two drugs, 1,5-isoquinolinediol (IQD) and ionic 4-cyano-*N*-indan-5-yl-benzamide (CIB) were predicted to have the potential to prevent or reverse senescence in all three tissues.

### *In vitro* validation test with murine embryonic fibroblasts (MEFs)

**Figure 4:**
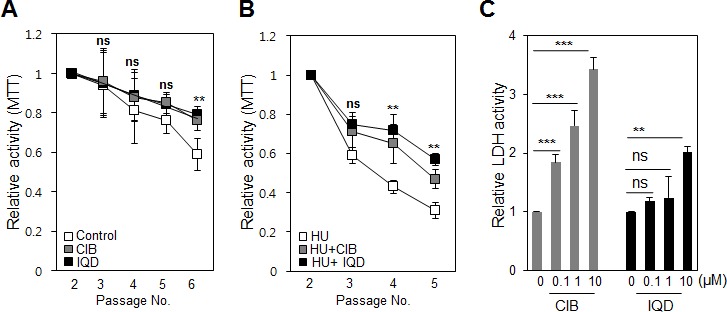
Testing changes in cell death and proliferation induced by the agents **A.** Murine embryonic fibroblasts (MEFs) were sub-cultured until passage number 5. The mitochondrial activity was assessed using an MTT assay in the presence or absence of the chemicals ionic 4-cyano-N-indan-5-yl-benzamide (CIB) and 1,5-isoquinolinediol (IQD). **B.** To induce oxidative stress and accelerate the senescence process, cells were pre-treated with hydroxyurea (HU) at 10 μM, and the anti-senescence effect of the chemicals was again tested using an MTT assay. **C.** To test the cytotoxicity of increasing concentrations of the drugs, cell death was measured by monitoring the lactate dehydrogenase (LDH) concentration in the culture media. ns, not significant; **, *p* < 0.01; ***, *p* < 0.001.

As the 3-(4,5-dimethylthiazol-2-yl)-2,5-diphenyltetrazolium bromide (MTT) assay measures the proliferation activity, we utilized it to conversely analyze the senescence rate, presuming that a decrease in the percent (%) value would reflect replicative senescence. We tested the anti-aging effect of CIB and IQD on murine embryonic fibroblasts (MEFs). As culture passage progressed, the proliferation rate, as determined by the MTT assay, gradually decreased in the control, but the decrease was not statistically significant. Treatment with CIB and IQD inhibited the decrease in proliferation. At passage number six, the inhibitory effect of CIB and IQD on senescence was particularly remarkable (Figure 4A). Hydroxyurea was used to accelerate the replicative senescence rate by inducing oxidative stress in MEFs. The proliferative activity showed a steeper decline compared with culture conditions without hydroxyurea. The anti-senescence effect of the two chemicals was more noticeable from passage number 3 (Figure 4B). The toxicity of CIB and IQD was then tested because a decreased MTT percent value can also be due to cell death. We tested the amount of lactate dehydrogenase (LDH) secreted into the culture media, and gradually increased the dose of CIB and IQD in an independent culture. While CIB induced cell death in proportion to the dose of the chemical, IQD did not significantly change the rate of cell death except at a dose of 10 μM (Figure 4C). Thus, only IQD was tested further.

### Anti-senescence effect of IQD

**Figure 5:**
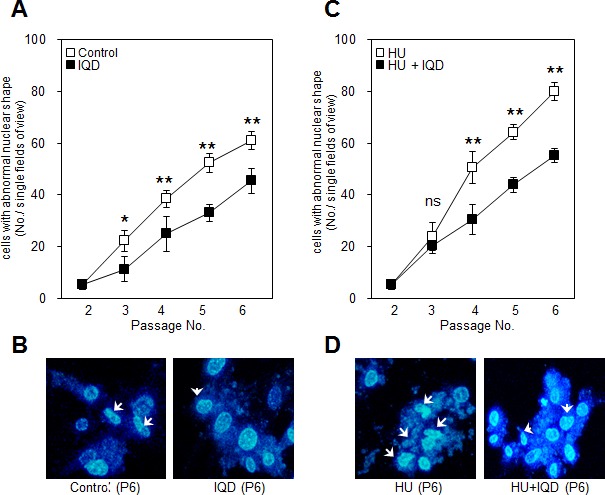
Effect of 1,5-isoquinolinediol (IQD) on preserving the shape of the nucleus **A.** With each passage, the shape of the nucleus was examined in the presence or absence of IQD. **B.** Representative images from different treatment conditions are displayed. White arrows indicates nuclei with an abnormal shape **C.** In the hydroxyurea (HU)-pretreated condition, aberrantly changed nuclei were counted using fluorescence microscopy after DAPI staining. **D.** Images indicate representative patterns.

**Figure 6:**
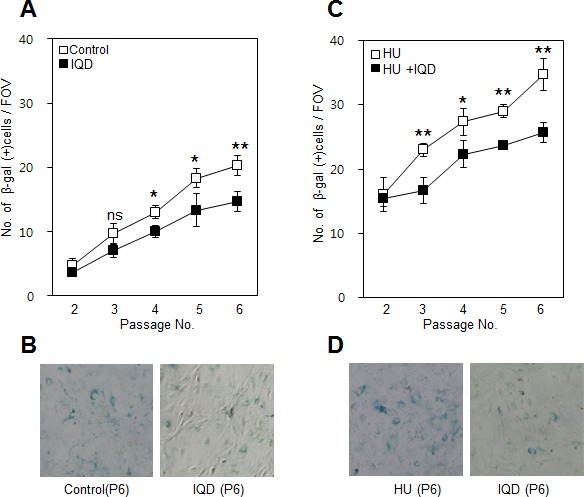
Effect of 1,5-isoquinolinediol (IQD) on formation of β-galactosidase **A.** Senescence was assessed using β-galactosidase staining after treatment with IQD. **B.** Lower panel shows representative field of view on microscopy at passage number 5. **C.** Under the senescence condition accelerated by hydroxyurea (HU), the number of β-galactosidase-positive cells was counted. **D.** Images showing representative β-galactosidase-positive cells. ns, not significant; *, *p* < 0.05; **, *p* < 0.01.

We examined the shapes of the nuclei for any changes corresponding to the number of passages. The cells showed a more abnormal nuclear shape as passaging progressed (Figure 5A and 5B). However, treatment with IQD suppressed the appearance of abnormal nuclei. Even under oxidative stress conditions, cells incubated with IQD presented fewer cells containing abnormal nuclei compared with untreated control cells (Figure 5C and 5D). We further tested the anti-senescence effect using β-galactosidase staining. Like the results for abnormally shaped nuclei, the number of β-galactosidase-positive cells increased noticeably as the passage number increased. When IQD was added, the increasing trend for β-galactosidase-positive cells was diminished (Figure 6A and 6B). Although hydroxyurea exacerbated the rate of cellular senescence, IQD significantly ameliorated the rate (Figure 6C and 6D).

### Anti-aging efficacy of IQD is related to decreased PARP1 activity

**Figure 7:**
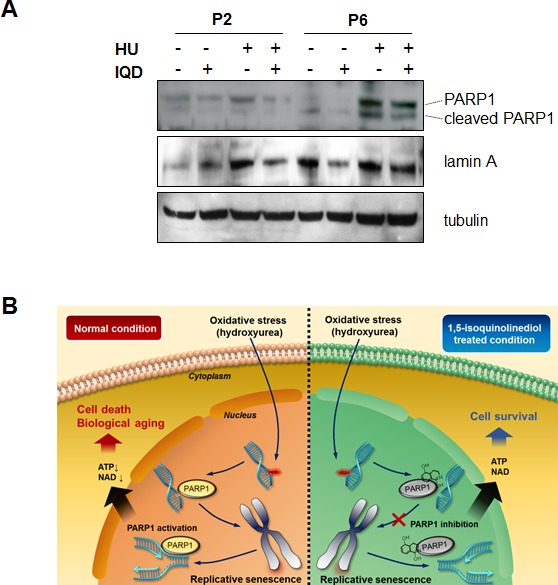
Inhibitory effect of 1,5-isoquinolinediol (IQD) on PARP1 activity **A.** Murine embryonic fibroblasts were treated with IQD at passage number 2 and 6. PARP1 was immunoblotted to verify the expression level, and the expression of lamin A was assessed to monitor replicative senescence. Tubulin was used as a loading control. **B.** Conceptual diagram to explain the anti-senescence effect of IQD. In basal replicative senescence conditions or oxidative stress, PARP1 is activated and is associated with biological aging in the different tissues. Treatment with IQD partially ameliorates the rate of senescence.

To confirm the inhibitory effect of IQD on PARP1, we selected MEFs from passage numbers 2 and 6 and treated them with IQD in the presence or absence of hydroxyurea. Hydroxyurea induced expression of PARP1 and lamin A, which is a protein marker for senescence (Figure 7). Treatment with IQD inhibited the induction of PARP1. At passage number 6, cleaved PARP1 appeared and its expression was also decreased by IQD.

Taken together, these results suggest that IQD could effectively suppress the senescence rate by decreasing the activity of PARP1 even under the conditions of oxidative stress induced by hydroxyurea in our experiment.

## DISCUSSION

According to recent statistics, aging is a risk factor in the pathogenesis of various diseases [21]Preventing or slowing aging may decrease the incidence of these diseases and ultimately extend the human life span. Although anti-aging drugs have already been introduced [22], we focused our attention on the fact that aging-related molecular mechanisms are basically different between tissues. As aging is a phenomenon that affects multiple tissues [23], to simultaneously address these tissue differences seemed to be critical for the development of more effective anti-aging drugs.

We applied a previously developed algorithm called a connectivity map to identify a potential new anti-aging drug [20]. The program enables us to predict a particular agent that induces a particular gene expression signature by linking a drug to gene expression or, conversely, gene expression to a drug. Here we input three aging-related gene expression signatures obtained from the endothelium, muscle cells, and neurons [22-24]. We did not perform microarray experiments ourselves, and instead collected the gene expression data from the GEO profile public website. Despite the limits imposed by using public data, information was obtained for three tissues, namely the artery, muscle, and cerebral cortex, which have importance in aging-associated diseases [21]. As we intended to find a universal anti-aging agent applicable to different tissues, instead cell lines derived from each tissues described above, MEFs, which are primary cells obtained from mice, was used to validate the anti-senescence effect. As the fibroblast has a slightly different function and gene expression pattern depending on its location [25], it is considered to reflect the varying genetic backgrounds of different organs.

Our bioinformatic analysis indicated IQD and CIB as anti-aging drugs. CIB showed toxicity in our initial screening. Thus, we continued investigating only the PARP inhibitor IQD. A decreased ability to repair DNA damage is related to the aging process, and can cause premature aging [26], neurodegeneration, and a loss of diversity in the stem cell compartment [27]. PARP1 is a well-known DNA repair enzyme. The PARP superfamily contains at least 17 enzymes that affect several biological processes such as transcriptional regulation, DNA repair, cell cycle regulation, the hypoxic response, and cell death [28]. Thus, a PARP inhibitor is logically likely to inhibit aging. PARPs perform poly(ADP-ribosyl)ation, which is drastic covalent posttranslational modification of proteins. This distinctive characteristic is an immediate cellular response to genotoxic insults induced by ionizing radiation, alkylating agents, and oxidative stress [28]. Increased activity of PARP1 consumes NAD^+^, which is required by SIRT1, thus causing SIRT1 to become relatively inactivated. As SIRT1 controls energy homeostasis, decreased activity of SIRT1 may result in oxidative stress and aging. Depletion of PARP1 increases NAD^+^ in the brown adipose tissue and muscle, thereby activating SIRT1 [29]. In general, PARP1 may work as a co-activator of nuclear factor-kappa B, which leads to chronic inflammation and increased oxidative stress [30]. The stress damages DNA and aggravates damage to the telomeres. A faster rate of telomere shortening induces cell senescence, which accelerates biological aging. Pharmacological inhibition of PARP could increase NAD^+^ levels, SIRT1 activity, and oxidative metabolism, leading to a decrease in oxidative stress and the prevention of aging [31].

Here, we suggest a potential anti-aging effect of the PARP1 inhibitor IQD that could simultaneously target several tissues. At a cellular level, inhibition of PARP1 seems to have an anti-senescence effect. This strategy may provide a novel antiaging therapy, and have clinical implications in the future.

## MATERIALS AND METHODS

### Analysis of gene expression with age

A linear mixed model was used to examine variability in gene expression by age, with confounding factors such as the batch and RNA concentration (only in skin samples) included as fixed effects, and the family relationship and zygosity included as random effects. We fitted the mixed-effects model in R with the lmer function in the lme4 package. The *p-*values to assess the significance of the age effect were calculated from the chi-square distribution with 1 degree of freedom using the likelihood ratio as the test statistic. The *p*-values adjusted for multiple testing were computed by controlling the false discovery rate (FDR) with the Benjamini-Hochberg procedure in R and using a threshold of 0.01. Enrichment analysis was carried out using the DAVID Bioinformatics Resource server with a threshold level of significance of 0.05 for Benjamini-Hochberg-corrected *p*-values.

### Pharmacogenomic analysis

To define the aging-related gene expression signature, the probe IDs from each tissue were merged into categories defined by gene symbols. Those gene symbols were separated into genes with 2.5-fold up- and down-regulated expression in the young and old groups for the connectivity map. The two groups of gene symbols were converted into the probe IDs of the Affymetrix HG U133A array for connectivity map analysis [20]. The top 40-ranked chemicals with statistical significance were selected for each tissue and the lists were compared with each other to detect overlaps.

### Cell and chemical preparation

Murine embryonic fibroblast (MEFs) were isolated from 12.5-day embryos of C57BL/6 mice and cultured in Dulbecco's modified Eagle medium (DMEM; Hyclone) containing 10% fetal bovine serum (FBS) and 2% penicillin/streptomycin. The cells from passage number 2 were used for all experiments. The chemical 1,5-isoquinolinediol (IQD, CID: 1340) and ionic 4-cyano-N-indan-5-yl-benzamide (CIB, CID : 668646) were purchased from ChemBridge(CA, US) and Cayman (MI, US), respectively.

### MTT assay

Cell growth was measured using an MTT assay (Promega, WI, US) according to the manufacturer's protocol. Briefly, cells were seeded in 96-well plates at a density of 5 × 10^3^ cells per well. After treatment with CIB or IQD (1 μmol/mL) in the presence or absence of pre-treatment with hydroxyurea (10 μmol/mL), the cells were incubated with 5 mg/mL MTT for 4 h. Then, the medium was removed and 150 μL of solubilization solution and stop solution was added, followed by incubation at 37 °C for 4 h. The absorbance of the reaction solution was measured at 570 nm. The cell growth inhibition rate was (1 − absorbance of experimental group/absorbance of control group) × 100%.

### LDH assay

After cells were seeded into 24-well plates at 5 × 10^4^ cells per well, they were treated with CIB and IQD (1 μmol/mL) for 1 to 7 days. The media were prepared separately. For the LDH assay, an LDH Cytotoxicity Assay kit (Cayman MI, US) was used as described by the manufacturer. Briefly, the cells were grown at 37 °C and 5% CO_2_ in DMEM supplemented with 10% heat-inactivated FBS and 1% penicillin/streptomycin. The cells were seeded into 96-well plates at a concentration of 2 × 10^4^ cells per well. After 48 h, 100 μL of the supernatant of the cultured cells was transferred from each well to the corresponding wells of a new plate, and 100 μL of reaction solution was added to each well. Plates were incubated with gentle shaking on an orbital shaker for 30 min at room temperature. The absorbance was read at 490 nm using a plate reader.

### DAPI staining

To observe the shape of the nucleus, MEFs were fixed with picric acid/formaldehyde for 20 min at room temperature and post-fixed with 70% ethanol. DAPI (Sigma Aldrich, MO, US) was diluted in cell culture medium and added at a final concentration of 10 ng/mL for 30 min at room temperature. DAPI-stained nuclei were individually traced by hand, and the size, length of the minor axis, length of the major axis, and shape factor were measured using Metamorph software (Universal Imaging).

### Western blot

MEFs were washed and solubilized with sodium dodecyl sulfate; proteins were separated on 10% polyacrylamide gels and then transferred to nitrocellulose membranes. The antibodies for PARP-1 (H-250) and laminA (H-102) were purchased from Santa Cruz Biotechnology Inc. (Dallas, TX, USA), and diluted to 1:1000 with 1% skim milk dissolved in TBS-T. Antibody binding was detected using the ECL Plus enhanced chemiluminescence system (Amersham Biosciences) with exposure of X-ray film.

### Senescence revealed by β-galactosidase (SA β-gal) staining

SA-β-gal staining was performed using the Senescence β-Galactosidase Staining Kit according to the manufacturer's instructions (Biovision, CA, US). Briefly, after washing with phosphate-buffered saline (PBS), cells were fixed with 2% formaldehyde and 0.2% glutaraldehyde in PBS for 15 min at room temperature. The fixed cells were incubated with X-gal staining solution at 37°C for 24 h after PBS washing. Blue colored cells were visualized. Using 200X objective lens of microscopy, the number of blue colored cells was counted in random four different fields of view (FOV)

## SUPPLEMENTARY MATERIAL TABLES



## References

[1] Falandry C, Bonnefoy M, Freyer G, Gilson E (2014). Biology of cancer and aging: a complex association with cellular senescence. Journal of clinical oncology : official journal of the American Society of Clinical Oncology.

[2] Pievani M, Filippini N, van den Heuvel MP, Cappa SF, Frisoni GB (2014). Brain connectivity in neurodegenerative diseases—from phenotype to proteinopathy. Nature reviews Neurology.

[3] Weinert BT, Timiras PS (2003). Invited review: Theories of aging. Journal of applied physiology.

[4] Lopez-Otin C, Blasco MA, Partridge L, Serrano M, Kroemer G (2013). The hallmarks of aging. Cell.

[5] Demontis F, Piccirillo R, Goldberg AL, Perrimon N (2013). Mechanisms of skeletal muscle aging: insights from Drosophila and mammalian models. Disease models & mechanisms.

[6] Yankner BA, Lu T, Loerch P (2008). The aging brain. Annual review of pathology.

[7] Johnson FB, Sinclair DA, Guarente L (1999). Molecular biology of aging. Cell.

[8] Glass D, Vinuela A, Davies MN, Ramasamy A, Parts L, Knowles D, Brown AA, Hedman AK, Small KS, Buil A, Grundberg E, Nica AC, Di Meglio P, Nestle FO, Ryten M, consortium UKBE (2013). Gene expression changes with age in skin, adipose tissue, blood and brain. Genome biology.

[9] Baker DJ, Wijshake T, Tchkonia T, LeBrasseur NK, Childs BG, van de Sluis B, Kirkland JL, van Deursen JM (2011). Clearance of p16Ink4a-positive senescent cells delays ageing-associated disorders. Nature.

[10] Collado M, Blasco MA, Serrano M (2007). Cellular senescence in cancer and aging. Cell.

[11] Brandes RP, Fleming I, Busse R (2005). Endothelial aging. Cardiovascular research.

[12] Dirks AJ, Hofer T, Marzetti E, Pahor M, Leeuwenburgh C (2006). Mitochondrial DNA mutations, energy metabolism and apoptosis in aging muscle. Ageing research reviews.

[13] Ziegler DV, Wiley CD, Velarde MC (2015). Mitochondrial effectors of cellular senescence: beyond the free radical theory of aging. Aging cell.

[14] Harrison DE, Strong R, Sharp ZD, Nelson JF, Astle CM, Flurkey K, Nadon NL, Wilkinson JE, Frenkel K, Carter CS, Pahor M, Javors MA, Fernandez E, Miller RA (2009). Rapamycin fed late in life extends lifespan in genetically heterogeneous mice. Nature.

[15] Kaeberlein M (2010). Resveratrol and rapamycin: are they anti-aging drugs?. BioEssays : news and reviews in molecular, cellular and developmental biology.

[16] Kennedy BK, Pennypacker JK (2014). Drugs that modulate aging: the promising yet difficult path ahead. Translational research : the journal of laboratory and clinical medicine.

[17] Barger JL, Kayo T, Vann JM, Arias EB, Wang J, Hacker TA, Wang Y, Raederstorff D, Morrow JD, Leeuwenburgh C, Allison DB, Saupe KW, Cartee GD, Weindruch R, Prolla TA (2008). A low dose of dietary resveratrol partially mimics caloric restriction and retards aging parameters in mice. PloS one.

[18] Markus MA, Morris BJ (2008). Resveratrol in prevention and treatment of common clinical conditions of aging. Clinical interventions in aging.

[19] Stanfel MN, Shamieh LS, Kaeberlein M, Kennedy BK (2009). The TOR pathway comes of age. Biochimica et biophysica acta.

[20] Lamb J, Crawford ED, Peck D, Modell JW, Blat IC, Wrobel MJ, Lerner J, Brunet JP, Subramanian A, Ross KN, Reich M, Hieronymus H, Wei G, Armstrong SA, Haggarty SJ, Clemons PA (2006). The Connectivity Map: using gene-expression signatures to connect small molecules, genes, and disease. Science.

[21] Kung HC, Hoyert DL, Xu J, Murphy SL (2008). Deaths: final data for 2005. National vital statistics reports : from the Centers for Disease Control and Prevention, National Center for Health Statistics, National Vital Statistics System.

[22] Marchand A, Atassi F, Gaaya A, Leprince P, Le Feuvre C, Soubrier F, Lompre AM, Nadaud S (2011). The Wnt/beta-catenin pathway is activated during advanced arterial aging in humans. Aging cell.

[23] Berchtold NC, Cribbs DH, Coleman PD, Rogers J, Head E, Kim R, Beach T, Miller C, Troncoso J, Trojanowski JQ, Zielke HR, Cotman CW (2008). Gene expression changes in the course of normal brain aging are sexually dimorphic. Proceedings of the National Academy of Sciences of the United States of America.

[24] Liu D, Sartor MA, Nader GA, Pistilli EE, Tanton L, Lilly C, Gutmann L, IglayReger HB, Visich PS, Hoffman EP, Gordon PM (2013). Microarray analysis reveals novel features of the muscle aging process in men and women. The journals of gerontology Series A, Biological sciences and medical sciences.

[25] Chang HY, Chi JT, Dudoit S, Bondre C, van de Rijn M, Botstein D, Brown PO (2002). Diversity, topographic differentiation, and positional memory in human fibroblasts. Proceedings of the National Academy of Sciences of the United States of America.

[26] Burkle A (2006). DNA repair and PARP in aging. Free radical research.

[27] Sahin E, Depinho RA (2010). Linking functional decline of telomeres, mitochondria and stem cells during ageing. Nature.

[28] Boesten DM, de Vos-Houben JM, Timmermans L, den Hartog GJ, Bast A, Hageman GJ (2013). Accelerated aging during chronic oxidative stress: a role for PARP-1. Oxidative medicine and cellular longevity.

[29] Bai P, Canto C, Oudart H, Brunyanszki A, Cen Y, Thomas C, Yamamoto H, Huber A, Kiss B, Houtkooper RH, Schoonjans K, Schreiber V, Sauve AA, Menissier-de Murcia J, Auwerx J (2011). PARP-1 inhibition increases mitochondrial metabolism through SIRT1 activation. Cell metabolism.

[30] Hassa PO, Hottiger MO (2002). The functional role of poly(ADP-ribose)polymerase 1 as novel coactivator of NF-kappaB in inflammatory disorders. Cellular and molecular life sciences : CMLS.

[31] Mangerich A, Burkle A (2012). Pleiotropic cellular functions of PARP1 in longevity and aging: genome maintenance meets inflammation. Oxidative medicine and cellular longevity.

