# Supplementary material for: Pharmacogenomic analysis indicates potential of 1,5-isoquinolinediol as a universal anti-aging agent for different tissues
Source: Oncotarget. 2015 Apr 29;6(19):17251–60. doi: 10.18632/oncotarget.3949 (PMC4627305; doi:10.18632/oncotarget.3949)
Supplement: Supplementary file 1 [file oncotarget-06-17251-s001.pdf]

Pharmacogenomic analysis indicates potential of 1,5-isoquinolinediol as a universal anti-aging agent for different tissues

Supplementary Material

Supplementary Table 1

Differentially expressed genes edited from GSE16487 were applied to connectivity map to obtain the candidate agents with potential to reverse aging of artery media. The top 40 ranked chemicals were listed up.

GSE16487		
rank	cmap name	enrichment score
1	thapsigargin	0.99
2	2-deoxy-D-glucose	0.983
3	tyrphostin AG-1478	0.982
4	(-)-catechin	0.973
5	MG-132	0.966
6	oligomycin	0.962
7	5149715	0.957
8	5140203	0.952
9	gefitinib	0.936
10	decitabine	0.935
11	5186324	0.902
12	5114445	0.89
13	arachidonyltrifluoromet	0.889
14	BW-B70C	0.885
15	PHA-00665752	0.882
16	anisomycin	0.877
17	topiramate	0.853
18	ambroxol	0.846
19	5182598	0.844
20	5152487	0.837
21	semustine	0.827
22	1	0.81
23	5666823	0.801
24	diperodon	0.777
25	dexverapamil	0.774
26	cefazolin	0.762
27	5162773	0.752
28	nimodipine	0.746
29	1,4-chrysenequinone	0.733
30	5109870	0.727
31	celastrol	0.719
32	mepacrine	0.717
33	phenoxybenzamine	0.714
34	dipivefrine	0.713
35	menadione	0.712
36	calmidazolium	0.706
37	astemizole	0.703
38	irinotecan	0.7
39	securinine	0.699
40	fenbendazole	0.699

Supplementary Table 2: Differentially expressed genes edited from GSE11882 were applied to connectivity map to obtain the candidate agents with potential to reverse aging of biceps brachii. The top 40 ranked chemicals were listed up.

**GSE38718**		
rank	cmap name	enrichment score
1	cytochalasin B	0.998
2	5149715	0.995
3	BW-B70C	0.992
4	2-deoxy-D-glucose	0.956
5	tomelukast	0.946
6	TTNPB	0.937
7	5140203	0.934
8	5109870	0.931
9	W-13	0.924
10	decitabine	0.904
11	5666823	0.884
12	dopamine	0.87
13	DL-PPMP	0.855
14	clemastine	0.832
15	penbutolol	0.821
16	hexetidine	0.821
17	cefoperazone	0.814
18	vigabatrin	0.813
19	lasalocid	0.812
20	viomycin	0.807
21	5186324	0.799
22	diloxanide	0.791
23	MS-275	0.79
24	Prestwick-692	0.783
25	5186223	0.763
26	HC toxin	0.762
27	naringenin	0.759
28	atractyloside	0.752
29	alfadolone	0.75
30	rifampicin	0.749
31	adiphenine	0.737
32	isoflupredone	0.733
33	atracurium besilate	0.73
34	podophyllotoxin	0.728
35	5151277	0.726
36	chenodeoxycholic acid	0.722
37	1,5-isoquinolinediol	0.721
38	scriptaid	0.715
39	levomepromazine	0.709
40	monensin	0.706

Supplementary Table 3: Differentially expressed genes edited from GSE38718 were applied to connectivity map to obtain the candidate agents with potential to reverse aging of superior frontal gyrus. The top 40 ranked chemicals were listed up.

**GSE11882**		
rank	cmap name	enrichment score
1	(-)-catechin	0.951
2	1,5-isoquinolinediol	0.948
3	MG-132	0.929
4	alcuronium chloride	0.909
5	sulfadiazine	0.905
6	STOCK1N-35874	0.895
7	exisulind	0.873
8	ketoconazole	0.865
9	5666823	0.859
10	natamycin	0.846
11	cantharidin	0.83
12	trazodone	0.817
13	DL-thiorphan	0.811
14	piperlongumine	0.806
15	8-azaguanine	0.801
16	verteporfin	0.775
17	dexverapamil	0.767
18	harmine	0.764
19	morantel	0.762
20	monocrotaline	0.76
21	scopoletin	0.757
22	ouabain	0.753
23	lisinopril	0.752
24	triflusal	0.736
25	carbachol	0.716
26	cefalexin	0.713
27	picotamide	0.708
28	noretynodrel	0.703
29	ursodeoxycholic acid	0.696
30	Prestwick-1084	0.693
31	etomidate	0.691
32	liothyronine	0.691
33	meticrane	0.689
34	tiabendazole	0.681
35	5252917	0.681
36	benzydamine	0.68
37	ipratropium bromide	0.68
38	valdecoxib	0.676
39	fenoterol	0.675
40	5114445	0.673
